# Supplementary material for: Complexome profiling of the Chlamydomonas psb28 mutant reveals TEF5 as an early PSII assembly factor
Source: Plant Cell. 2025 Mar 17;37(6):koaf055. doi: 10.1093/plcell/koaf055 (PMC12164590; doi:10.1093/plcell/koaf055)
Supplement: koaf055_Supplementary_Data [file koaf055_supplementary_data.zip › psb28_interactive.zip!/plots/intensity/qualityPlots/peptides/_Cre09.g416850.t1.2.html]

### Cre09.g416850.t1.2

Trivial name:   
SeqCoverage [median]: 16.10  
Mass: 165.19 kDa  
  
 

| Sequence | MissedCleavage | Mass [Da] | wtIntensity | muIntensity | Score |
| --- | --- | --- | --- | --- | --- |
| AAAQVYR | 0 | 777.41 | 798.66 | 8580.94 | 187.48 |
| AAEALSR | 0 | 716.38 | 0.00 | 1211.61 | 82.67 |
| AATAAASADASAVNR | 0 | 1345.66 | 269.65 | 3136.30 | 130.01 |
| AGQTAFSAAGGAASAAAK | 0 | 1506.74 | 324.52 | 2585.54 | 80.71 |
| ALDLVDAVGDAVVK | 0 | 1383.76 | 2744.15 | 0.00 | 103.34 |
| ALLLDVR | 0 | 798.50 | 0.00 | 2855.04 | 73.04 |
| ALPTGVRPSAK | 1 | 1095.64 | 569.37 | 4871.32 | 63.41 |
| AVVGPDVDVEAAAADPSALAEAAGR | 0 | 2321.15 | 1402.12 | 4165.33 | 121.47 |
| EKEVGATAAAADPR | 1 | 1384.69 | 0.00 | 368.28 | 39.03 |
| GAVAGLTGAAAELPR | 0 | 1352.74 | 4220.29 | 25791.77 | 148.28 |
| GEVVPEGLVVPPALVAK | 0 | 1672.98 | 794.39 | 0.00 | 45.08 |
| GGAMPAASAGGGGVDAAGGAPR | 0 | 1753.82 | 0.00 | 1140.92 | 43.12 |
| LTGLPVMDPPEPAAEAR | 0 | 1762.89 | 0.00 | 1456.89 | 56.20 |
| NSGDVYEQGLADLRPFRR | 2 | 2092.05 | 0.00 | 2369.20 | 27.98 |
| SAADAASVATNLSASR | 0 | 1490.73 | 880.64 | 9111.98 | 203.39 |
| TLAEAVAAEGSAR | 0 | 1244.64 | 4650.71 | 16418.94 | 135.66 |
| VLAVGAVGGVGVAAASR | 0 | 1452.84 | 272.53 | 1045.41 | 84.61 |
| VLLLDSYGVEAPEAVALLR | 0 | 2027.13 | 462.59 | 0.00 | 121.28 |
|  |  |  | 17389.6 | 85109.5 |  |
